# Mapping the diverse trajectories of intrinsic capacity and their key drivers in China’s aging population

**DOI:** 10.3389/fpubh.2026.1923924

**Published:** 2026-10-15

**Authors:** Ziyi Liang, Zhirou Liang, Linlin Xie, Keqing Liang, Jianxiong Hu, Sujuan Chen, Tao Liu, Jiagui Liang, Guanhao He, Fan Wu, Wenjun Ma

**Affiliations:** 1 Department of Public Health and Preventive Medicine, School of Medicine, Jinan University, Guangzhou, China; 2 School of Public Health, Fudan University, Shanghai, China; 3 China Greater Bay Area Research Center of Environmental Health, School of Medicine, Jinan University, Guangzhou, China

**Keywords:** China, healthy aging, influencing factors, intrinsic capacity, trajectories

## Abstract

**Background:**

Intrinsic capacity (IC) is central to healthy aging. Understanding its longitudinal change is crucial for proactive health planning. Yet evidence on distinct IC trajectories and their determinants in China remains limited, constraining targeted interventions.

**Methods:**

We analyzed cohort data from participants aged 50 years and older in the World Health Organization’s Study on Global AGEing and Adult Health (WHO-SAGE) China cohort. A composite IC score was constructed based on the WHO framework, encompassing locomotion, cognition, psychological, sensory, and vitality domains. We employed Latent Class Growth Analysis (LCGA) and Growth Mixture Modeling (GMM) to identify distinct IC trajectories. Multinomial logistic regression was then used to investigate associations between these trajectories and a comprehensive set of socio-demographic, individual health-related, and environmental factors. The relative importance of identified factors was evaluated using SHapley Additive exPlanations (SHAP) values.

**Results:**

Four IC trajectories were identified: High and Stable IC (HSI), High and Slowly Declining IC (HSDI), Moderate and Rapidly Declining IC (MRDI), and Low and Rapidly Declining IC (LRDI). Older age (OR = 5.79, 95% CI 4.73–7.10), female sex (OR = 2.86, 95% CI 2.18–3.75), comorbidity (OR = 8.15, 95% CI 5.07–13.10), low income (OR = 4.11, 95% CI 2.82–5.99), low paternal education (OR = 2.04, 95% CI 1.41–2.94), and higher physical risk scores (OR = 1.18, 95% CI 1.02–1.37) were associated with a greater likelihood of belonging to the LRDI trajectory and were also associated with other unfavorable profiles. Clean cooking fuel use (OR = 0.17, 95% CI 0.11–0.26) and being married (OR = 0.53, 95% CI 0.34–0.82) were associated with a lower likelihood of belonging to the LRDI trajectory. SHAP identified age as the most influential contributor across all trajectories, while income, cooking fuel, ambient temperature, relative humidity, sex, education, and comorbidity also showed substantial contributions across different trajectory patterns.

**Conclusion:**

The study identified substantial heterogeneity in IC trajectories among Chinese middle-aged and older adults. Socio-demographic, individual health-related, and environmental factors were associated with distinct trajectory patterns. Potentially modifiable contextual factors, particularly household cooking fuel and environmental conditions, may warrant greater attention in future healthy ageing strategies.

## Introduction

The global population is aging at an accelerating pace, presenting a major challenge to public health and social systems worldwide and placing unprecedented strain on healthcare and long-term care infrastructures (1). Nowhere is this demographic shift more pronounced than in China, which is home to the world’s largest population of older adults—a number that continues to grow rapidly. It is projected that by 2050, more than one-third of China’s population will be aged 60 or older (2). This trend not only intensifies the societal burden of chronic disease but also underscores the urgent need to promote healthy aging and improve the quality of life in later years—a cornerstone of sustainable human development.

In response, the WHO has introduced the concept of intrinsic capacity (IC) as an innovative and comprehensive indicator of healthy aging (3). Defined as the composite of an individual’s physical and mental capacities, IC integrates five core domains: locomotion, cognition, vitality, psychological wellbeing, and sensory function (4). Unlike conventional disease-oriented metrics, IC offers a dynamic and person-centered perspective, capturing the trajectory of functional change and providing a holistic view of an aging individual’s reserves and risks (5). A growing body of evidence associates low IC with increased incidence of chronic diseases, injuries, and mortality (6–10). More recent longitudinal studies further suggest that patterns of IC decline—particularly rapid deterioration during the transition from midlife to older age—can serve as early warning signals for subsequent adverse health outcomes (11–13). Thus, tracking IC trajectories may provide important insights into heterogeneity in the ageing process and changes in individual capacity over time.

This study was framed within the WHO Healthy Ageing framework, which defines healthy ageing as the process of developing and maintaining the functional ability that enables wellbeing in older age. Functional ability reflects the combined influence of an individual’s intrinsic capacity, the environments in which the individual lives, and the interaction between them. Within this framework, intrinsic capacity represents the composite of an individual’s physical and mental capacities and constitutes a central individual-level component of healthy ageing (3, 4). Importantly, IC is dynamic rather than static and may vary substantially across individuals over the ageing process (5). Socioeconomic conditions, health status, and environmental contexts may therefore be associated with heterogeneous patterns of IC change over time. Guided by this framework, the present study focused on identifying longitudinal IC trajectories and examining the multidimensional factors associated with these distinct patterns, with particular attention to socioeconomic, health-related, and environmental contexts.

Despite its conceptual promise, empirical research on IC trajectories, particularly studies adopting a multidimensional perspective, remains limited. Several longitudinal studies from different populations, including cohorts such as the English Longitudinal Study of Ageing (ELSA) (14), the Health and Retirement Study (HRS) (15), and the Mexican Health and Aging Study (MHAS) (16), have explored patterns of IC change over time. However, existing evidence has several limitations. First, many previous studies have primarily examined selected categories of factors, such as sociodemographic characteristics or clinical conditions, while environmental exposures (e.g., ambient temperature and household fuel type) and broader contextual factors have received comparatively less attention. Second, few studies have systematically evaluated the relative contribution of multidimensional factors to different IC trajectories. Third, variations in trajectory modeling approaches and limited characterization of heterogeneous ageing patterns have restricted a comprehensive understanding of IC evolution. Given the dynamic nature of IC across the ageing process, identifying factors associated with different IC trajectories is important for understanding heterogeneity in functional ageing. Existing literature suggests that IC is associated with socio-demographic characteristics (e.g., age, sex, education, and income) (17, 18), health-related factors (e.g., comorbidities) (19, 20), and environmental exposures (21). Therefore, an integrated framework combining longitudinal trajectory modeling with multidimensional assessment is needed to better understand heterogeneous patterns of IC change.

Furthermore, although regression models are valuable for identifying independent associations and estimating their direction and magnitude, they are less suited for directly comparing the relative contributions of factors across diverse domains. Assessing variable importance can provide additional insights into which factors contribute most substantially to heterogeneous IC trajectories. Therefore, interpretable machine learning approaches, such as SHAP, may complement traditional regression analyses by providing an intuitive assessment of relative variable importance and contribution patterns.

To address these gaps, this study leverages data from the China cohort of the WHO-SAGE to: (1) identify distinct trajectories of intrinsic capacity among Chinese middle-aged and older adults; (2) investigate multidimensional factors spanning socio-demographic, health-related, and environmental domains that are associated with different IC trajectories; and (3) quantify the relative contributions of these factors across different trajectory patterns. Our findings are expected to provide evidence for understanding heterogeneity in IC development and to inform population-level strategies aimed at supporting healthy ageing.

## Methods

### Study settings and population

All participants were from the SAGE-China cohort, a prospective cohort study conducted in six countries (China, Ghana, India, Mexico, Russia, and South Africa) (22). The Chinese SAGE cohort was carried out in eight provincial-level administrative regions (Jilin, Zhejiang, Shandong, Guangdong, Hubei, Shaanxi, Yunnan, and Shanghai). Data for this dynamic cohort study were drawn from three waves of the WHO Study on Global AGEing and Adult Health (SAGE) China: Wave 1 (2007–2010), Wave 2 (2014–2015), and Wave 3 (2018–2019). From an initial pool of 18,673 participants across the three waves, participants without available follow-up information, those younger than 50 years at baseline, and those with missing key variables were excluded. After these exclusions, 5,067 participants were included in the final analysis (Supplementary Figure S1).

### Data collection

To facilitate the present study, all participants entering the cohort underwent face-to-face interviews at baseline, during which demographic characteristics, basic health status, and lifestyle behaviors were collected. In the subsequent two survey waves, data collection included both follow-up of previously enrolled participants and recruitment of new respondents, with the same standardized questionnaires as those used at baseline. For the purposes of this analysis, we restricted the study population to individuals who completed all three survey waves, with full information available at baseline, the first follow-up, and the second follow-up, to ensure the reliability of longitudinal comparisons and trend analyses. Ethical approval for this study was obtained from the Ethics Committee of the Chinese Center for Disease Control and Prevention (Approval No. 200601) and the Shanghai Municipal Center for Disease Control and Prevention (Approval No. 2014-8 and 2018-1). Written informed consent was obtained from all participants prior to the surveys.

### Intrinsic capacity

This survey draws on the World Health Organization’s (WHO) conceptual framework of Intrinsic Capacity (IC). As no universally standardized IC measurement tool has been established for large-scale epidemiological studies, we operationalized IC by mapping available SAGE items onto the five core domains of the WHO IC framework. The selected indicators corresponded to mobility, vitality, cognition, psychological status (depression), and sensory function (23). The composite IC score demonstrated acceptable internal consistency in our sample (standardized Cronbach’s *α* = 0.70).

Each domain was measured using one or two items reflecting participants’ subjective functional status over the past 30 days. Specifically, mobility was assessed by difficulty with daily movement; vitality by feeling tired or lacking energy during the day; cognition by difficulty learning new tasks (e.g., navigating new places or acquiring new skills); psychological status by feelings of sadness or depression; and sensory function by vision (e.g., difficulty reading) and hearing (e.g., difficulty hearing others clearly in a quiet room). All items were rated on a five-point scale (1 = no difficulty, 5 = extreme difficulty or inability to perform) and reverse-coded so that higher scores indicated better functional status and higher IC levels. The total IC score was calculated as the sum of all item scores. Detailed information on the domain-item mapping and scoring is provided in Supplementary Table S1. To minimize potential circularity, the specific items used to construct the IC score were not reused as predictors, although some predictors were conceptually related to IC domains but represented distinct measurements.

### Potential factors

Based on previous studies (17–21), we included factors from three dimensions: (1) socio-demographic factors; (2) individual health-related factors; (3) environmental factors. The variables included in each dimension are listed in Supplementary Tables S2–S4.

Socio-demographic variables included age, sex, marital status, education (parents’ and own), urbanization, income, and GDP per capita. Age was self-reported. Sex was self-reported and referred to sex assigned at birth (male/female). Marital status was dichotomized as “married” (currently married or cohabiting) and “unmarried” (never married, separated, divorced, or widowed). Urbanization (urban/rural) was self-reported. Parental and individual education levels were jointly categorized. Household income was divided according to the median annual family income at each survey wave, and GDP per capita (GDPpc) was similarly categorized based on the median GDP for each period. Detailed definitions are provided in Supplementary Table S2.

Individual health-related factors included physical factors, comorbidity and fall. Physical factors included BMI, grip strength, pulmonary function (FEV₁/FVC), pulse rate, and pre-hypertension (Supplementary Table S3). Within each domain, unfavorable and favorable statuses were coded as 1 and 0, respectively, and the five components were summed to generate a physical score ranging from 0 to 5. A higher score indicated a greater number of unfavorable physical factors. Comorbidity was defined as having ≥2 of seven chronic diseases (hypertension, diabetes, depression, asthma, stroke, angina, chronic lung disease) occurring within the past year (Supplementary Table S4). Fall history within the past year was coded as “yes” if participants reported ≥1 fall or fall-related injury, and “no” otherwise.

Environmental factors in this study included cooking fuel type (clean vs. unclean), ambient temperature, humidity, and PM₂.₅ concentration. To assess the associations of temperature, humidity, and PM₂.₅ with intrinsic capacity, daily meteorological data were obtained from the ERA5-Land reanalysis dataset, a global gridded product provided by the European Centre for Medium-Range Weather Forecasts (ECMWF). ERA5-Land provides near-surface air temperature and dew point temperature (°C) at a spatial resolution of 0.1° × 0.1° (approximately 10 km × 10 km) and has been widely applied in climate and health research (24). Daily relative humidity (%) was subsequently calculated using the daily mean temperature and dew point temperature following standard meteorological formulas (25). Daily mean PM₂.₅ concentrations (1 km × 1 km resolution) were extracted from the China High Air Pollutants (CHAP) dataset (http://www.geodata.cn), which has been validated against ground-based observations (R^2^ = 0.92, RMSE = 10.76 μg/m^3^) (26). Following previous studies (27), ambient PM_2.5_, temperature, and relative humidity were assigned to participants at the county/district level. Environmental grid cells within each study county or district were identified and spatially averaged, and the resulting county/district-level estimates were assigned to participants according to their residential county/district. For each participant, long-term environmental exposure was characterized using mean PM_2.5_, ambient temperature, and relative humidity over the follow-up period corresponding to IC trajectory assessment. Detailed definitions are provided in Supplementary Table S2.

Missing data were handled using a sequential approach. Participants with missing intrinsic capacity scores were excluded. For missing covariates, available information from adjacent waves was used whenever possible. Remaining missing covariate values (<5%) were imputed using multiple imputation by chained equations (MICE).

### Statistical analysis

Continuous variables were described as mean and standardized deviation, while categorical variables were described as frequency and proportion. To identify the heterogeneous longitudinal trajectories of IC, we performed Latent Class Growth Analysis (LCGA) and Growth Mixture Modeling (GMM) using the lcmm package in R (28). The model fitting followed a three-step step-up procedure (29): (1) an LCGA model, assuming fixed effects within classes, was first established to explore the basic latent class structure; (2) a GMM with random intercepts was then constructed to allow for inter-individual variation in baseline IC levels; and (3) a GMM with both random intercepts and random slopes was fitted to capture individual differences in baseline levels and rates of change over time. To reduce the risk of convergence to local maxima, a grid-search strategy with 100 random initial values was applied to the mixture models (28). For each model specification, we systematically evaluated 1–5 latent classes. Model selection was based on multiple complementary criteria, including lower Bayesian Information Criterion (BIC), higher entropy, adequate class size, model convergence, and theoretical interpretability of the trajectory patterns (30, 31). A class proportion of at least 5% was considered acceptable.

Given the potential of more than two IC trajectories, multinomial logistic regression were used to investigate the associations of socio-demographic, individual health-related, and environment factors on IC trajectory. The favorable trajectory (high IC and stable trajectory) was used as the reference group. The formula was presented as follow (32):


$$\text{logit}P_{k}=\ln[\frac{P(y=k\;|\;x)}{P(y=0\;|\;x)}]=\alpha_{k}+\beta_{k1}x_{1}+\beta_{k2}x_{2}+\cdots +\beta_{\mathrm{kp}}x_{p}$$

k=1,2,⋯,K−1

In this model, P(y=k∣x) represents the conditional probability of an individual belonging to category *k*, while P(y=0∣x) denotes the probability of the same individual belonging to the reference category under the same conditions. Here, $\alpha_{k}$ is the intercept term, $x_{1}$ , $x_{2}$ ,…, $x_{p}$ denotes the covariates, and $\beta_{k1}$ , $\beta_{k2}$ ,…, $\beta_{\mathrm{kp}}$ represents the regression coefficients, Multinomial logistic regression was incorporated to assess their overall effects.

To further characterize factors associated with IC trajectories, an interpretable machine learning framework was applied following multinomial logistic regression. Regression was used to estimate the direction and magnitude of associations with trajectory membership, whereas machine learning was used to evaluate the relative contribution of individual factors rather than for prediction. Physical factors were represented by a composite score in the regression to provide a domain-level estimate, but were disaggregated into their constituent variables in the primary machine learning analysis to enable factor-level assessment. Candidate factors were screened using repeated LASSO stability selection over 100 stratified 70% resamples, with the penalty parameter determined by five-fold cross-validation using the one-standard-error rule. Variables selected in at least 70% of repetitions were retained for machine learning analysis. Four algorithms—random forest (RF), support vector machine (SVM), naive Bayes (NB), and extreme gradient boosting (XGBoost)—were integrated into a stacking ensemble model. SHAP analysis was used to quantify the relative importance and direction of individual factor contributions to IC trajectory classification.

### Sensitivity analysis

To assess the robustness of the multinomial logistic regression results, sensitivity analyses were conducted using a block-wise approach. The main model simultaneously included socio-demographic, individual health-related, and environmental factors. Two additional models were fitted by excluding one covariate domain at a time: (i) Model A included environmental and socio-demographic factors; and (ii) Model B included environmental and individual health-related factors. These models were used to assess whether the observed associations were robust to different combinations of covariate domains.

In addition, a machine learning sensitivity analysis was performed using the composite physical score rather than its individual components. The same LASSO stability-selection procedure and ensemble stacking framework as in the primary analysis were applied to assess whether domain-level, rather than component-level, representation of physical factors materially affected the overall variable importance patterns.

We followed the STROBE reporting guideline in preparing this manuscript. The completed STROBE checklist is submitted as a separate file as required by the journal.

## Results

### The characteristics of participants

A total of 5,067 participants were included in this study, including 2,519 males (49.71%). Urban residents accounted for 62.92%, while 67.00% were from southern China. Generational education patterns revealed notable parent–child differences: mother–child pairs were predominantly characterized by “generational exchange” (59.23%), followed by “stably low” (35.80%), while “stably high” was rare (4.97%); similar patterns were also observed in father-child pairs; other characteristics at baseline were presented in Supplementary Table S5. Environmental data during follow-up showed a mean temperature of 14.72 ± 4.10 °C, humidity of 72.52 ± 4.81%, and PM₂.₅ concentration of 48.49 ± 12.30 μg/m^3^. Detailed information was shown in Supplementary Table S6.

During follow-ups, the proportion of participants aged ≥70 years increased markedly from 19.44% wave 1(baseline) to 49.97% at wave 3. We also observed significant change of marital status, with unmarried participants (never married, separated, divorced, or widowed) increasing from 12.61 to 21.69%. The prevalence of comorbidity rose from 10.54% at wave 1 to 32.66% at wave 2. Clean fuel usage increased from 50.15 to 67.18%. The physical score increased from 1.43 ± 0.93 at Wave 1 to 1.52 ± 0.95 at Wave 3. Other characteristics are presented in Table 1.

**Table 1:** Characteristics of the study cohort (*N* = 5,067) at each survey wave.

Characteristics	Wave 1 (Baseline) (2007–2010)	Wave 2 (2014–2015)	Wave 3 (2018–2019)
Age, *n* (%)			
50–59 years	2,420(47.76%)	906(17.88%)	89(1.76%)
60–69 years	1,662(32.80%)	2,360(46.58%)	2,446(48.27%)
≥70 years	985(19.44%)	1801(35.54%)	2,532(49.97%)
Marital status, *n* (%)			
Married	4,428(87.39%)	4,230(83.48%)	3,968(78.31%)
Unmarried	639(12.6%)	837(16.52%)	1,099(21.69%)
Fall, *n* (%)			
Yes	147(2.90%)	71(1.40%)	195(3.85%)
No	4,920(97.10%)	4,996(98.60%)	4,872(96.15%)
Comorbidity, *n* (%)			
Yes	534(10.54%)	969(19.12%)	1,655(32.66%)
No	4,533(89.46%)	4,098(80.88%)	3,412(67.34%)
Cooking fuel, *n* (%)			
Clean	2,541(50.15%)	2,969(58.59%)	3,404(67.18%)
Non-clean	2,526(49.85%)	2098(41.41%)	1,663(32.82%)
GDP per capita, *n* (%)			
Low	2,385(47.07%)	2,464(48.63%)	2,412(47.60%)
High	2,682(52.93%)	2,603(51.37%)	2,655(52.40%)
Physical risk score,			
Mean±SD	1.43 ± 0.93	1.47 ± 0.91	1.52 ± 0.95

(1) For participants and their father’s (mother’s) education, stably high means both parents and the participant at least completed primary school; stably low means both parents and the participant did not complete primary education; generational change represents that parents did not complete primary education, while the participant completed primary school, or parents at least completed primary education while the participant did not complete primary education. (2) The physical score was calculated based on BMI status, grip strength, lung capacity, pulse rate, and pre-hypertension. Details are provided in Supplementary Table S3. (3) The same 5,067 participants were included in all three waves; differences across waves reflect within-cohort changes over time rather than differences in participants.

### The characteristics of IC and its functional domains

IC shows age-dependent reductions across its five functional domains—cognition, mobility, vitality, sensory function, and depression, with reductions becoming increasingly rapid in older adults (Figure 1a, Supplementary Figures S2, S3). Similar patterns were observed in both men and women across all IC domains, with men consistently maintaining higher IC levels than women across ages. Among the five domains, cognition exhibited the lowest mean levels across waves in both sexes (Figure 1b). Cross-generational analysis confirmed a universal age-related IC decline across the 1930s–1950s birth cohorts, with a distinct cohort-specific pattern: at the same age, participants born in the 1950s exhibited lower IC levels than those born in the 1930s or 1940s (Figure 1c).

**Figure 1:**
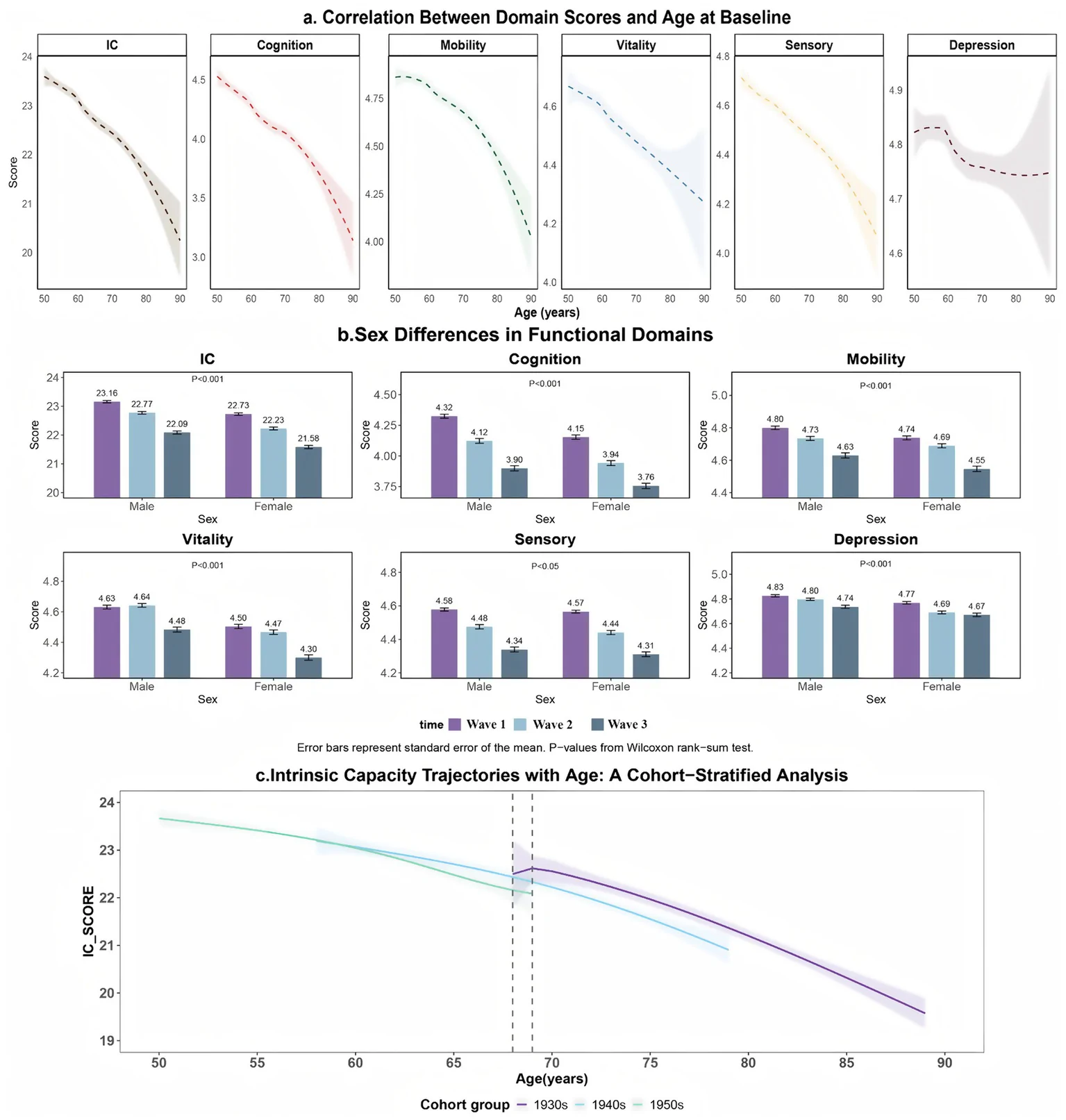
The scores of IC and its functional domain by age, sex and birth cohort. **(a)** The age trajectories of scores for IC and its functional domains at baseline survey; **(b)** The comparisons of IC and its functional domains between wave 1, wave 2 and wave 3 by sex; **(c)** The comparisons of age trajectories of IC among 1930s, 1940s and 1950s birth cohorts. The higher the score, the healthier it is.

### The trajectories of IC

Through integrated evaluation of BIC, statistical robustness, class balance, theoretical interpretability, and class proportions, We identified four heterogeneous IC trajectories: high and stable IC (HSI) showed relatively high baseline scores and stable IC over time; high and slowly declining IC (HSDI) maintained high baseline IC with minimal decline; moderate and rapidly declining IC (MRDI) had moderate IC but declined fast; and low and rapidly declining IC (LRDI) had the lowest baseline scores with steeply decline (Figure 2, Supplementary Table S7). Sex-stratified analyses demonstrated similar trajectory patterns in both men and women. For LRDI and MRDI trajectories, age-stratified analyses revealed that older people demonstrated sharper decline patterns than youngers (Figure 2). We further observed similar decline patterns of IC across regions, urbanization, and comorbidity status (Supplementary Figures S4–S6).

**Figure 2:**
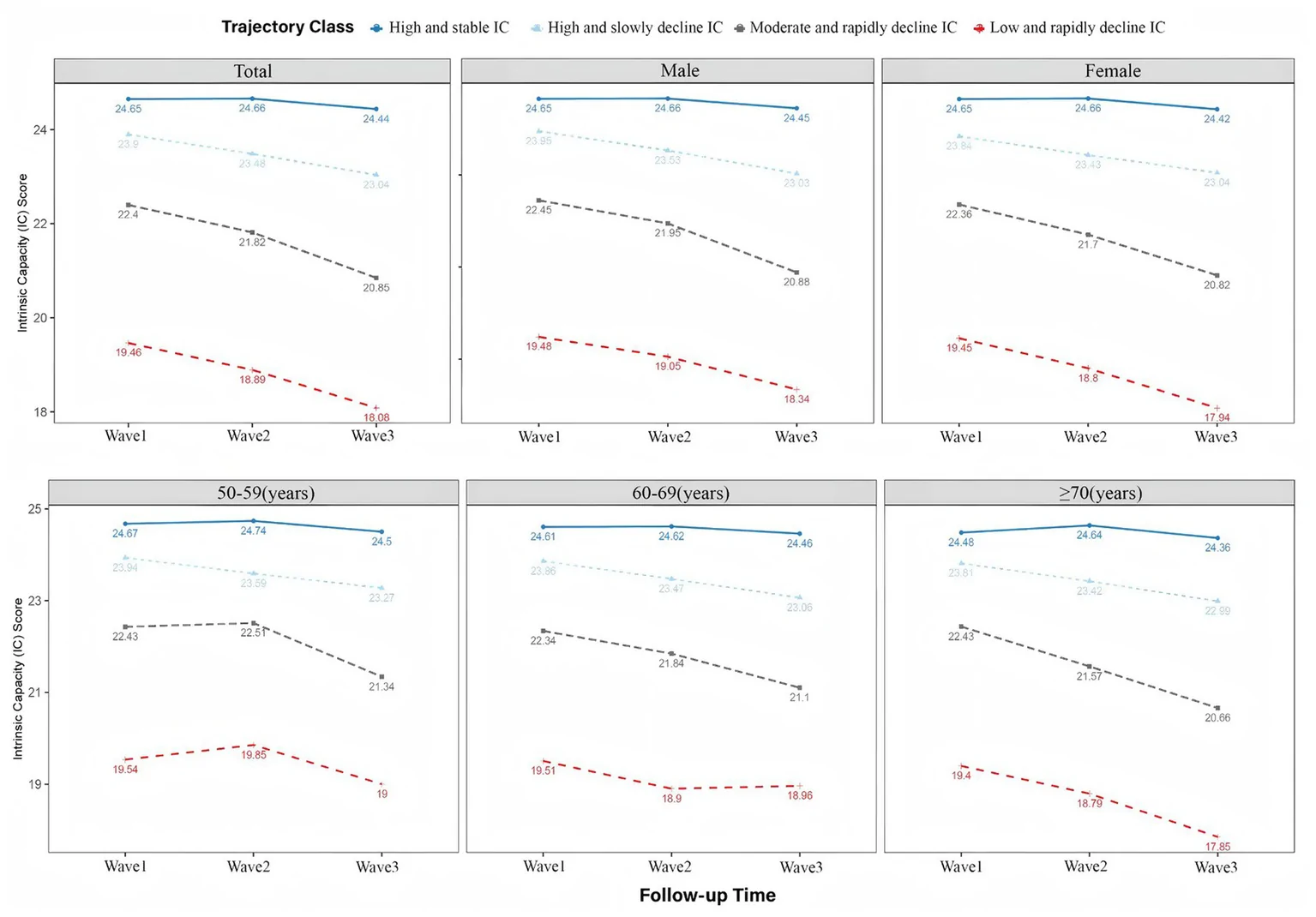
The IC trajectories from wave 1 to wave 3 by sex, age in the WHO SAGE China cohort.

### Factors associated with IC trajectories

Supplementary Table S8 summarizes baseline socio-demographic, individual health-related, and environmental characteristics across the four IC trajectories, with significant overall differences observed among trajectory groups. Preliminary diagnostics (Supplementary Table S9) showed that humidity had an extremely high VIF due to shared variance with several climatic and regional socioeconomic variables. After excluding the region variable, multicollinearity was substantially reduced (Supplementary Tables S9, S10). Using HSI as the reference group, multinomial logistic regression showed that older age (OR = 5.79, 95% CI 4.73–7.10), female sex (OR = 2.86, 95% CI 2.18–3.75), comorbidity (OR = 8.15, 95% CI 5.07–13.10), low income (OR = 4.11, 95% CI 2.82–5.99), and low paternal education (OR = 2.04, 95% CI 1.41–2.94) were associated with higher odds of belonging to the LRDI trajectory, and these factors were also associated with other unfavorable trajectories such as HSDI and MRDI. Higher physical risk scores (OR = 1.18, 95% CI 1.02–1.37) were associated with higher odds of belonging to the LRDI trajectory, whereas clean fuel use (OR = 0.17, 95% CI 0.11–0.26) and being married (OR = 0.53, 95% CI 0.34–0.82) were associated with lower odds, and similar associations were observed across the HSDI and MRDI trajectories. Temperature did not show a consistent significant association across trajectory comparisons (Figure 3).

**Figure 3:**
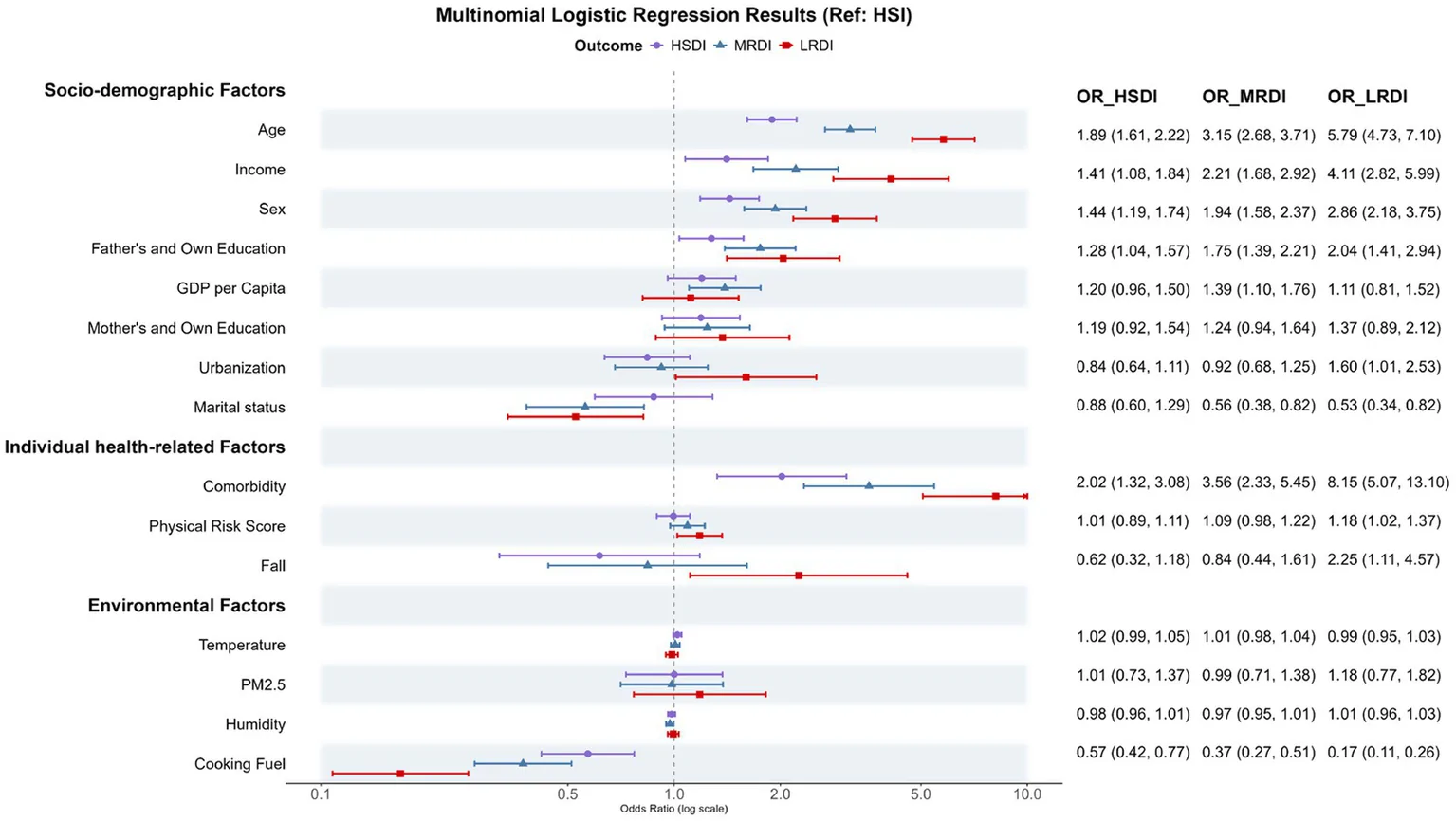
The socio-demographic, individual health-related and environmental factors of IC trajectories (HSDI, MRDI, LRDI) compared with HSI trajectory from wave 1 to wave 3. Odds ratios (ORs) are shown with 95% confidence intervals (CIs). Temperature and humidity represent the mean values during the follow-up period. Reference groups were defined as follows: sex = male; comorbidity = no; fall = no; marital status = unmarried; urbanization = urban; income = high; GDP per capita = high; cooking fuel = non-clean; PM₂.₅ = non-good. Continuous variables (age, father’s (mother’s) and own education, physical risk score, humidity, and temperature) were modeled per one-unit increase.

Sensitivity analyses were performed to verify the robustness of these associations by re-estimating the models under different combinations of covariate domains. The direction and magnitude of the associations remained stable. Core factors such as age, comorbidity, income, and fuel type consistently showed associations across sensitivity models (Supplementary Table S11).

### SHAP analysis of IC trajectories

To further quantify the relative importance of individual factors associated with IC trajectories, we used SHapley Additive exPlanations (SHAP) based on an ensemble stacking model integrating RF, SVM, NB, and XGBoost. Prior to the SHAP analysis, the physical risk score was disaggregated into its constituent variables for factor-level assessment, and multicollinearity was examined. No substantial collinearity was observed (Supplementary Table S12).

Age consistently ranked as the most influential factor across all four trajectories. Income also showed high relative importance, ranking second for HSI, HSDI, and MRDI. Cooking fuel, ambient temperature, relative humidity, sex, and educational indicators also contributed substantially, particularly for the HSDI and MRDI trajectories. For the LRDI trajectory, comorbidity ranked second in importance after age, followed closely by income. Fall status generally showed relatively low importance across trajectories. Stacking-based mean absolute SHAP values are presented in Figure 4, and results from the four individual base learners are provided in Supplementary Figures S7–S11.

**Figure 4:**
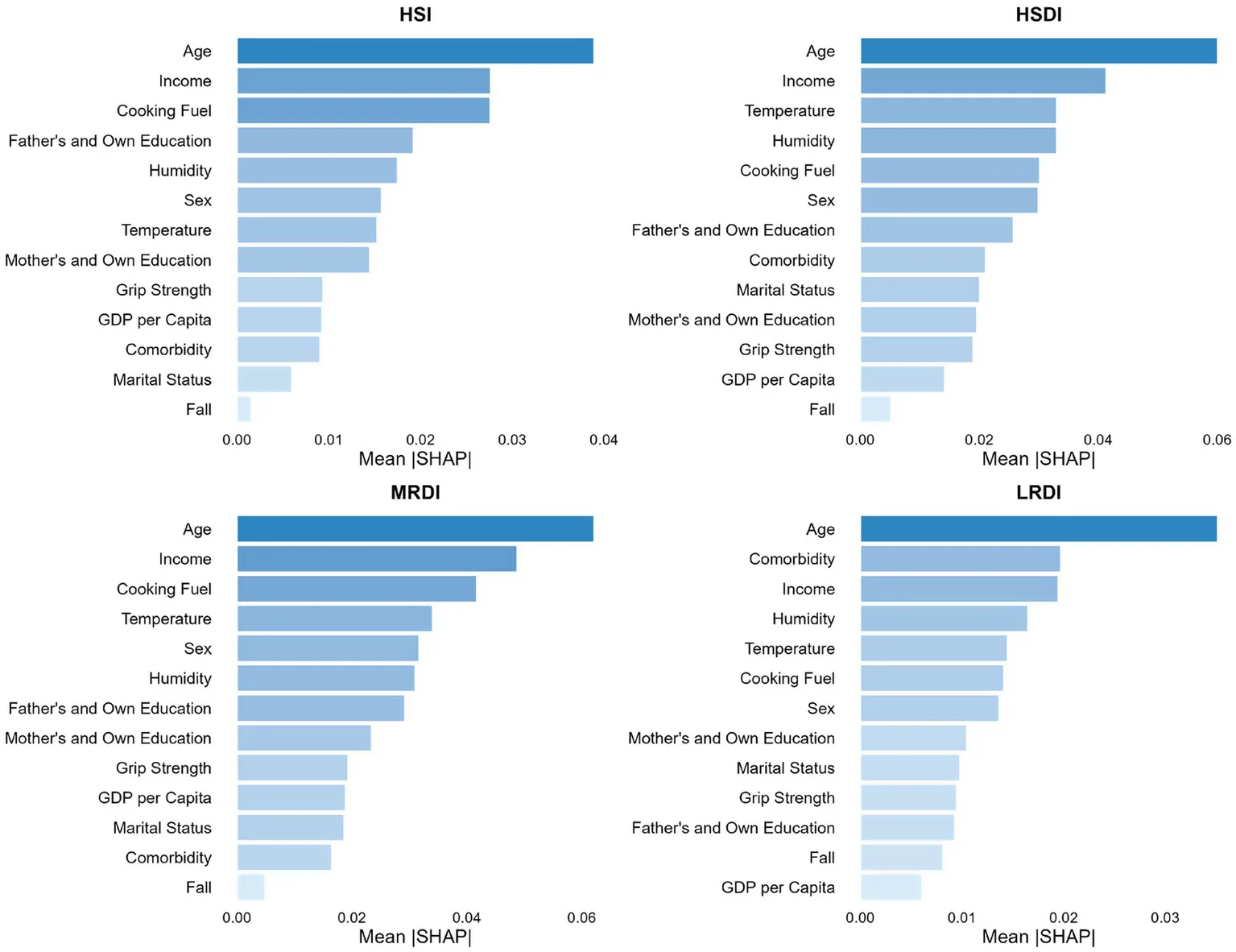
Feature importance based on stacking meta-learner for different IC trajectories.

A sensitivity analysis using composite physical scores instead of their disaggregated components yielded broadly consistent feature-selection and importance patterns. Age, income, education, comorbidity, cooking fuel, and ambient temperature remained among the major contributors, supporting the robustness of the primary findings. Corresponding results from the stacking model and individual base learners are presented in Supplementary Figures S12–S17.

To further characterize the direction of individual factor contributions, stacking-weighted signed SHAP summary plots were generated (Figure 5). Higher age values showed predominantly negative SHAP values for HSI and HSDI and positive SHAP values for MRDI and LRDI. Low income, lower educational attainment, female sex, comorbidity, and unhealthy grip strength showed similar contrasting patterns, tending to contribute away from HSI and HSDI and toward MRDI and LRDI. In contrast, clean cooking fuel and being married tended to contribute toward HSI and HSDI and away from MRDI and LRDI. Environmental factors showed trajectory-specific directional patterns: higher relative humidity generally contributed toward HSI and HSDI and away from MRDI and LRDI, whereas higher ambient temperature contributed toward HSI and HSDI and away from MRDI, with no clear directional pattern for LRDI. Overall, the signed SHAP distributions demonstrated distinct contribution patterns across the four IC trajectories.

**Figure 5:**
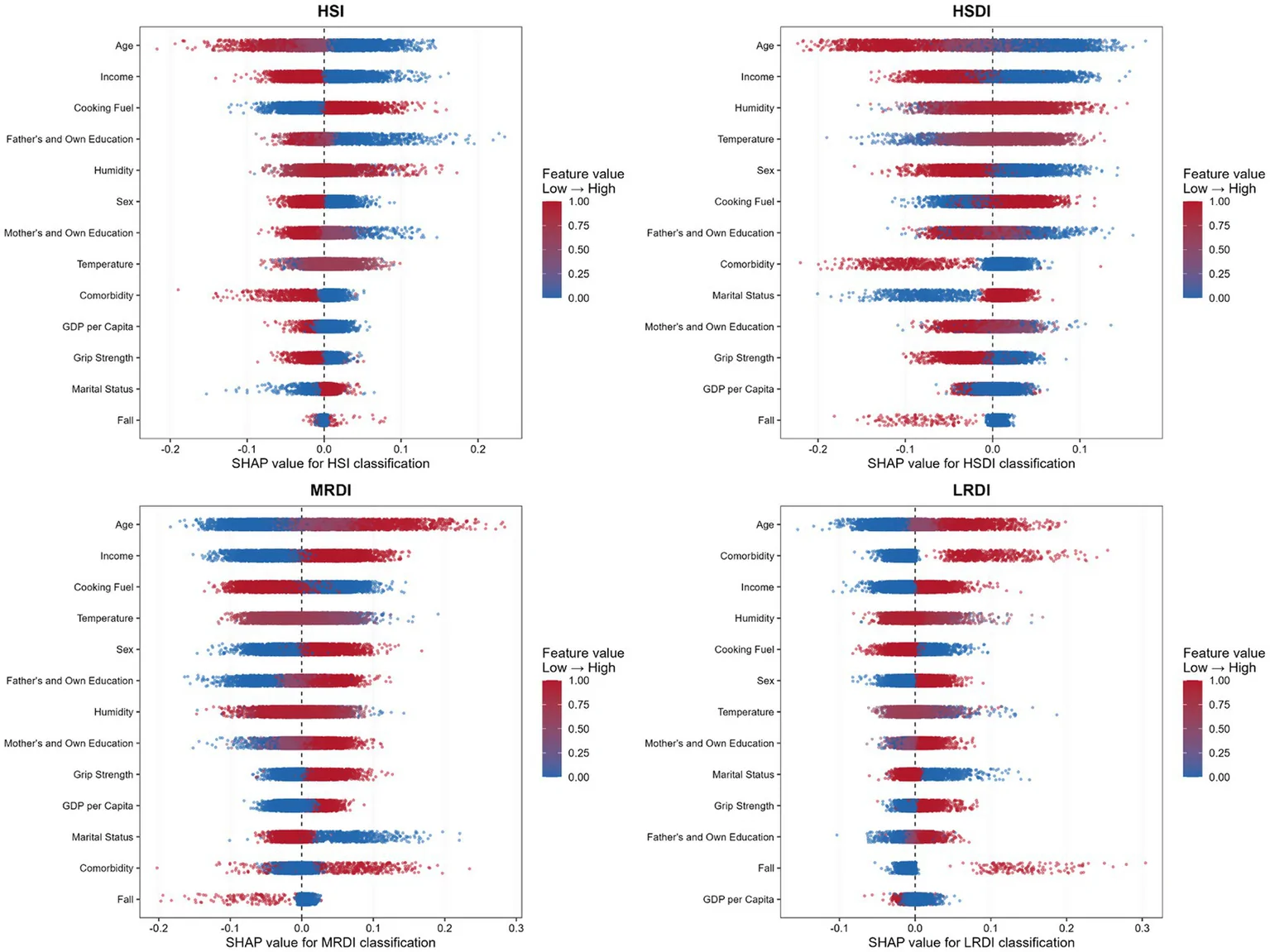
Directional contributions of factors to IC trajectory classification based on stacking-weighted signed SHAP values. Higher values of paternal and maternal education scores indicate lower educational attainment.

## Discussion

Based on a large cohort of Chinese middle-aged and older adults, this study examined longitudinal changes in IC and its five domains and found that IC declined progressively with age, with a more rapid decline observed among older adults. We further identified four distinct IC trajectories: HSI, HSDI, MRDI, and LRDI. Older age, female sex, comorbidity, low income, and use of unclean household fuels were associated with a greater likelihood of belonging to unfavorable trajectories, whereas better physical function, being married, and use of clean household fuels were associated with more favorable trajectory patterns. SHAP analysis further identified age as the most influential contributor across trajectories, with income, cooking fuel, ambient temperature, relative humidity, sex, education, and comorbidity also showing substantial contributions across different trajectory patterns. These findings highlight population groups and contextual factors associated with unfavorable IC trajectories and may help inform future healthy ageing strategies.

We observed a continuous decline in IC when age rises, with a more pronounced decrease at advanced ages (12, 33, 34). This is in line with the findings from both the United Kingdom and China (35). These findings indicate that IC decline reflects the multisystem nature of aging, characterized by cumulative losses in musculoskeletal strength, sensory function, and neurocognitive capacity, accompanied by chronic inflammation, oxidative stress, and reduced physiological resilience (36–38). Moreover, we observed a cohort-specific pattern wherein the 1950s-born cohort exhibited lower IC levels than earlier-born cohorts at the same age. While this pattern is consistent with life-course perspectives such as the Developmental Origins of Health and Disease framework, early-life adversity, including historical nutritional deprivation during periods such as the Great Chinese Famine, may represent one possible explanation (39–41). However, this interpretation should be viewed cautiously because age, period, and cohort effects are inherently intertwined in observational data. Future life-course studies specifically designed to disentangle APC effects are needed to clarify these potential mechanisms.

We identified four progression trajectories of IC among Chinese middle-aged and old populations, which is similar to previous studies (42, 43). For instance, Zhou et al (42) analyzed data from 2,454 adults aged 60 years and above in the Chinese Longitudinal Healthy Longevity Survey (CLHLS) and identified four IC trajectories: low IC with slow decline (3.2%), moderate IC with increase (13.0%), moderate IC with decline (12.0%), and stable high IC (71.8%). A study on older adults in Taiwan Province of China also reported similar IC trajectories (43). By lowering the age threshold from 60 to 50 years, our study extended the previous studies on IC trajectories to an earlier stage of aging, which allows capturing midlife functional transitions that may precede clinically apparent decline. The heterogeneity of IC progression may reflect differences in socioeconomic conditions, health status, and environmental contexts. Identifying individuals with rapidly declining trajectories may help characterize groups requiring greater clinical and supportive attention.

While previous studies have established associations between environmental, socioeconomic, and biological factors and healthy ageing or IC status, evidence regarding their roles in longitudinal IC trajectories remains limited. Our study addresses this gap by integrating environmental, socioeconomic, and individual health-related factors within a longitudinal trajectory framework and applying interpretable machine-learning approaches to evaluate their relative importance in a large Chinese cohort. We found that age, income, sex, cooking fuel, and comorbidity were among the influential factors associated with unfavorable IC trajectories. Older adults were more likely to exhibit rapidly declining patterns, consistent with previous studies on IC deterioration (44, 45). Similar to the findings of Salinas-Rodríguez (46), women were more likely than men to follow low or declining IC trajectories, possibly due to hormonal changes after menopause, longer life expectancy, and higher comorbidity burden (47). Chronic diseases may be associated with accelerated functional deterioration (48), consistent with prior longitudinal and mechanistic studies linking multimorbidity to accelerated declines in IC (49). Better physical function was associated with a lower likelihood of unfavorable IC trajectories, consistent with previous evidence linking physical capacity to slower deterioration of IC (50, 51). Lower income was significantly associated with a greater likelihood of belonging to low or rapidly declining trajectories, in line with previous studies (52). This highlights the potential importance of socioeconomic conditions for functional maintenance in later life. In addition, lower inter-generational education levels, reflected by paternal education as a marker of early-life family SES (53), have been associated with later-life functional decline (54, 55). This suggests that early-life socioeconomic disadvantage may be related to lower physiological and cognitive reserves in later life. Being married was also associated with more favorable trajectory patterns, potentially through emotional, social, and material support, consistent with findings from both Chinese and Western cohorts (56, 57). Taken together, these findings underscore the importance of socioeconomic conditions, chronic disease burden, physical function, and contextual factors in understanding heterogeneous patterns of IC change.

Environmental exposures also played an important role in shaping IC trajectories. Higher ambient temperature and household fuel type were associated with different IC trajectory patterns. These findings align with previous evidence linking household air pollution and ambient temperature variations to increased risks of cardiopulmonary dysfunction, cognitive decline, and accelerated aging (58, 59). Household fuel type reflects chronic exposure to indoor pollutants, and ambient temperature may influence IC through both behavioral and physiological pathways—such as reduced outdoor activity, impaired thermoregulation, and elevated cardiometabolic stress—ultimately compromising systemic resilience in later life (60, 61). Environmental conditions may therefore represent an important contextual factor shaping functional aging trajectories. Maintaining IC may therefore be related not only to individual health status but also to broader socioeconomic resources and living environments. Individuals exposed to solid fuels could be prioritized in clean energy transition initiatives, while reducing household air pollution may represent a potential strategy for improving environmental conditions associated with unfavorable functional aging trajectories.

To further characterize the relative importance of factors associated with IC trajectories, we applied interpretable machine-learning approaches with SHAP analysis. Beyond the associations identified in the regression analyses, our trajectory-based analysis further showed that the relative contributions of these factors varied across IC patterns. Age consistently showed the greatest contribution across all trajectory classifications, while socioeconomic and environmental factors, including income, household cooking fuel, ambient temperature, and relative humidity, also showed substantial contributions. Educational indicators were important across several trajectories, whereas comorbidity showed a particularly pronounced contribution to the LRDI trajectory, indicating that the relative importance of health-related factors differed according to the pattern of IC change. Grip strength also contributed to trajectory classification, consistent with previous evidence linking preserved muscle function with maintenance of intrinsic capacity (62, 63). Sensitivity analysis using the composite physical score rather than its individual components yielded broadly consistent feature-selection and variable-importance patterns, further supporting the robustness of the main findings. Overall, the SHAP findings demonstrate that the relative importance and direction of socioeconomic, health-related, and environmental factors varied across heterogeneous IC trajectories. These findings may help identify population groups and contextual factors warranting greater attention in future healthy ageing strategies.

These findings can also be interpreted within the WHO Healthy Ageing framework. In this framework, healthy ageing depends on functional ability, which reflects the interaction between an individual’s intrinsic capacity and the environment in which the individual lives. The heterogeneous IC trajectories observed in our study therefore illustrate different patterns of change in individual capacity over time, while the observed associations with socioeconomic, health-related, and environmental factors highlight the broader contexts in which these trajectories occur. This perspective emphasizes ageing as a dynamic and multidimensional process rather than solely as the accumulation of disease or functional deficits.

### Strengths and limitations of the study

Our study has several strengths. First, it integrates socio-demographic, individual, and environmental factors within a unified analytical framework to comprehensively examine factors associated with IC trajectories. Second, the longitudinal design across three survey waves enables dynamic modeling of IC decline over time. Third, the ensemble stacking framework ensures methodological rigor while preserving model interpretability via SHAP analysis. Fourth, this study has incorporated environmental indicators into IC modeling, providing a novel perspective for future research exploring the interplay between intrinsic capacity and environmental factors. However, Several limitations should be acknowledged. First, multiple imputation of missing data may have introduced some uncertainty. Second, environmental exposures were assigned at the county/district level rather than using exact residential coordinates, and information on residential mobility between survey waves was unavailable; both factors may have resulted in exposure misclassification. Third, because environmental exposures were averaged over the same general follow-up period in which IC trajectories were assessed, temporal precedence could not be established, limiting causal interpretation. Fourth, self-reported information and on-site measurements may be subject to recall, interviewer, and measurement biases. Finally, the absence of a universally standardized IC measurement tool may limit cross-study comparability.

## Conclusion

Our analysis identified four distinct IC trajectories among Chinese middle-aged and older adults. Older age, comorbidity, lower socioeconomic status, and lower educational attainment were associated with a greater likelihood of belonging to unfavorable trajectories. Potentially modifiable contextual factors, particularly household cooking fuel and environmental conditions, were also associated with distinct trajectory patterns. These findings highlight population groups and contextual factors that may warrant greater attention and provide evidence to inform population-level strategies for promoting healthy ageing within the broader healthy ageing framework.

## Data Availability

The datasets analyzed during the current study are not publicly available due to privacy restrictions but are available from the corresponding author on reasonable request.
