# Supplementary material for: Mapping the diverse trajectories of intrinsic capacity and their key drivers in China’s aging population
Source: Front Public Health. 2026 Oct 15;14:1923924. doi: 10.3389/fpubh.2026.1923924 (PMC13716573; doi:10.3389/fpubh.2026.1923924)
Supplement: Supplementary file 1 [file Data_Sheet_1.DOCX]

**Mapping the Diverse Trajectories of Intrinsic Capacity and their Key Drivers in China's Aging Population**

**Author:** Ziyi Liang^a+^, B.D, Zhirou Liang^b+^, B.D, Linlin Xie^a^, B.D, Keqing Liang^a^, B.D, Jianxiong Hu^a^, PhD, Sujuan Chen^a^, PhD, Tao Liu^a,d^, PhD, Jiagui Liang^a^, PhD, Guanhao He^a,d*^, PhD, Fan Wu^c**^, PhD, Wenjun Ma^a,d***^, PhD.

**Supplementary Materials**

**Contents**

[Figure S1. The flowchart of sample selection	3](#_Toc6965)

[Figure S2. The age trajectories of intrinsic capacity(IC) scores for each functional domain at follow-upI survey	4](#_Toc3971)

[Figure S3. The age trajectories of intrinsic capacity(IC) scores for each functional domain at follow-upII survey	4](#_Toc25677)

[Figure S5. The IC trajectories from baseline survey to follow-up II stratified by urbanization in the WHO SAGE China cohort	6](#_Toc7746)

[Figure S6. The IC trajectories from baseline survey to follow-up II stratified by comorbidity in the WHO SAGE China cohort	7](#_Toc7964)

[Figure S7. SHAP-based feature contributions across four base learners (class HSI)	8](#_Toc18768)

[Figure S8. SHAP-based feature contributions across four base learners (class HSDI)	9](#_Toc22937)

[Figure S9. SHAP-based feature contributions across four base learners (class MRDI)	10](#_Toc31872)

[Figure S10. SHAP-based feature contributions across four base learners (class LRDI)	11](#_Toc22330)

[Figure S11. SHAP-based feature contributions of the stacking meta-learner across intrinsic capacity trajectories	12](#_Toc170)

[Figure S12. SHAP-based feature contributions across four base learners (composite scores, class HSI)	13](#_Toc2988)

[Figure S13. SHAP-based feature contributions across four base learners (composite scores, class HSDI)	14](#_Toc30752)

[Figure S14. SHAP-based feature contributions across four base learners (composite scores, class MRDI)	15](#_Toc24583)

[Figure S15. SHAP-based feature contributions across four base learners (composite scores, class LRDI)	16](#_Toc24442)

[Figure S16. SHAP-based feature contributions of the stacking meta-learner (composite scores)	17](#_Toc16092)

[Figure S17. Feature importance of the stacking meta-learner across intrinsic capacity trajectories(composite scores)	18](#_Toc15862)

[Table S1. Definitions of Intrinsic Capacity Measurement Items included in this study	19](#_Toc1432)

[Table S2. Definitions of covariates included in this study[Total]	20](#_Toc18894)

[Table S3. Definitions of Physical Factor included in this study	22](#_Toc11787)

[Table S4. Definitions of Comorbidity Factor included in this study	23](#_Toc10277)

[Table S5. The characteristics of study participants	24](#_Toc22805)

[Table S6. Environmental characteristics during the follow-up period	25](#_Toc28030)

[Table S7. Comparison of Fit Statistics for (LCGA) in Intrinsic Capacity Trajectories (1–5 Classes)	26](#_Toc27588)

[Table S8. Distribution of Intrinsic Capacity Trajectories and Baseline Characteristics among middle aged and old people	27](#_Toc27361)

[Table S9. Preliminary multicollinearity diagnostics of all candidate variables before model selection	29](#_Toc14957)

[Table S10. Multicollinearity diagnostics of variables included in the multinomial logistic regression model	30](#_Toc29967)

[Table S11. Results of sensitivity analyses	31](#_Toc22919)

[Table S12. Multicollinearity diagnostics of predictors used in the machine learning analysis	32](#_Toc32377)

18,673 participants were included at baseline in this study from 2007-2018

- 15,050 participants were included in first wave (baseline investigation)

- 3,623 participants were included in second wave and third wave (follow-up investigations)

6619 participants with missing follow-up investigations’ information were excluded

6449 participants with missing complete three following investigations’ information were excluded

3 participants with missing residential district

11935 participants with completed information were included

5067 participants were included in the study

67 participants with missing Intrinsic capacity score were excluded

416 participants younger than 50 years old were excluded

Figure S1. The flowchart of sample selection

Figure S2. The age trajectories of intrinsic capacity(IC) scores for each functional domain at follow-upI survey

Figure S3. The age trajectories of intrinsic capacity(IC) scores for each functional domain at follow-upII survey

Figure S4. The IC trajectories from baseline survey to follow-up II stratified by region in the WHO SAGE China cohort

Figure S5. The IC trajectories from baseline survey to follow-up II stratified by urbanization in the WHO SAGE China cohort

Figure S6. The IC trajectories from baseline survey to follow-up II stratified by comorbidity in the WHO SAGE China cohort

Figure S7. SHAP-based feature contributions across four base learners (class HSI)

Figure S8. SHAP-based feature contributions across four base learners (class HSDI)

Figure S9. SHAP-based feature contributions across four base learners (class MRDI)

Figure S10. SHAP-based feature contributions across four base learners (class LRDI)

Figure S11. SHAP-based feature contributions of the stacking meta-learner across intrinsic capacity trajectories

Figure S12. SHAP-based feature contributions across four base learners (composite scores, class HSI)

Figure S13. SHAP-based feature contributions across four base learners (composite scores, class HSDI)

Figure S14. SHAP-based feature contributions across four base learners (composite scores, class MRDI)

Figure S15. SHAP-based feature contributions across four base learners (composite scores, class LRDI)

Figure S16. SHAP-based feature contributions of the stacking meta-learner (composite scores)

**Figure S17.** Feature importance of the stacking meta-learner across intrinsic capacity trajectories(composite scores)

Table S1. Definitions of Intrinsic Capacity Measurement Items included in this study

| **Domain** | **Question(In the last 30 days)，all the responses are in the range of 1 to 5 (none = 1, mild = 2, moderate = 3, severe = 4, extreme/cannot do = 5)** |
| --- | --- |
| Mobility | how much difficulty did you have with moving around? |
| Vitality | how much difficulty did you have due to not feeling rested and refreshed during the day (for example, feeling tired, not having energy)? |
| Cognition | how much difficulty did you have in learning a new task (for example, learning how to get to a new place, learning a new game, learning a new recipe)? |
| Depression | how much difficulty did you have with feeling sad, low or depressed? |
| Sensory | How much difficulty did you have in seeing and recognising an object at arm's length (for example, reading)? |
|  | How much difficulty did you have in hearing and understanding what two people were saying in a quiet room? |

Note: While in data processing, the scores of each dimension are assigned in reverse. The higher the IC score, the healthier it is.

Table S2. Definitions of covariates included in this study[Total]

| **Type** | **Variance** | **Definition** | | |
| --- | --- | --- | --- | --- |
| Individual health-related factors | Physical Factor | Detailed information is shown in Supplement Table S4. | | |
|  | Comorbidity | Detailed information is shown in Supplement Table S5. | | |
|  | Fall | Yes: Had experienced at least one fall or reported a fall-related injury;  No: No fall history or fall-related injury reported. | | |
| Socioeconomic Factors | Age | Age was self-reported | | |
|  | Sex | Sex was self-reported and referred to sex assigned at birth.  “Male”  “Female” | | |
|  | Marriage | “Married” included currently married or cohabiting.  “Unmarried” included never married，separated，divorced，and widowed. | | |
|  | Urbanicity | Urban_rural was self-reported.  “Urban”  “Rural” | | |
|  | Mother's Education & Own Education |  | Mother's Education | Participant’s Education |
|  |  | stable high | ≥primary | ≥primary |
|  |  | generational change | <primary | ≥primary |
|  |  |  | ≥primary | <primary |
|  |  | stable low | <primary | <primary |
|  | Father's Education & Own Education |  | Father's Education | Participant’s Education |
|  |  | stable high | ≥primary | ≥primary |
|  |  | generational change | <primary | ≥primary |
|  |  |  | ≥primary | <primary |
|  |  | stable low | <primary | <primary |
|  | Income |  | Division according to the median annual family income (Every time of survey). | |
|  | GDP per capita |  | Division according to the median GDP per capita（Every time） | |
| Environmental Factors | Fuel type |  | “Clean” included liquefied petroleum gas，natural gas, and electricity.  “Unclean” included kerosene, paraffin, coal, charcoal, wood, agriculture crop, dung, shrubs and grass. | |
|  | Temperature (℃) |  | Mean daily temperature over the follow-up period. | |
|  | Relative Humidity (%) |  | Mean daily relative humidity over the follow-up period. | |
|  | PM_2.5_ (μg/m^3^) |  | Mean daily PM_2.5_ concentration over the follow-up period. For regression analyses, PM_2.5_ was dichotomized according to the Chinese Ambient Air Quality Standard[GB 3005-2012] (Grade II annual mean limit, 35 μg/m³):  “Good”: <35 μg/m³)  “Poor”: ≥35 μg/m³) | |

Table S3. Definitions of Physical Factor included in this study

| **Variance** | **Definition** |
| --- | --- |
| BMI | Healthy: 18.5 ≤ BMI < 25  Unhealthty: Underweight, overweight, or obese |
| Pulmonary Function | Healthy: FEV1/FVC ≥70%  Unhealthty: FEV1/FVC <70% |
| Pulse | Healthy: 60–100 beats per minute  Unhealthty: <60 or >100 bpm |
| Pre_hypertension^[1]^ | Healthy: normotensive or hypertensive  Unhealthty: 120 ≤ SBP < 140 mmHg or 80 ≤ DBP < 90 mmHg |
| Grip strength^[2]^ | Healthy: Male ≥28 kg, Female ≥18 kg  Unhealthty: Male <28 kg, Female <18 kg |

Notes: The variance against with guidelines and recommendations were coded as “1”, and the “Physical risk score” variable was calculated as the sum of five factors, with a total scores ranging from 0 to 5.

[1]JUN C, YINGXIAN S, YUMING L, et al. Clinical practice guidelines for the management of hypertension in China [J]. Chinese Journal of Cardiology, 2024, 52(9): 985-1032.

[2]Chen LK, Woo J, Assantachai P, et al. Asian Working Group for Sarcopenia: 2019 Consensus Update on Sarcopenia Diagnosis and Treatment. J Am Med Dir Assoc. 2020;21(3):300-307.e2. doi:10.1016/j.jamda.2019.12.012

Table S4. Definitions of Comorbidity Factor included in this study

| **Variance** | **Definition** |
| --- | --- |
| Hypertension | Defined as having hypertension by self-report, medication use, or elevated blood pressure measurement. |
| Diabetes | Defined as having diabetes by self-report, medication use, diet control, or hospitalization for diabetes. |
| Depression | Defined as having depression by self-report, medication use, or meeting diagnostic criteria for major depressive episode. |
| Asthma | Defined as having asthma by self-report or medication/treatment use. |
| Stroke | Defined as having stroke by self-report, medication/treatment use, or hospitalization. |
| Angina pectoris | Defined as having angina by self-report or medication/treatment use. |
| Chronic lung disease | Defined as having chronic lung disease (e.g., COPD, bronchitis, emphysema) by self-report or medication/treatment use. |
| Comorbidity | Yes: had **≥2** of the above 7 diseases  No: had**＜2** of the above 7 diseases |

**Table S5. The characteristics of study participants**

| **Characteristics** | **n(%)** |
| --- | --- |
| **Sex** |  |
| Male | 2,519（49.71%） |
| Female | 2,548（50.29%） |
| **Urbanization** |  |
| Urban | 3,188（62.92%） |
| Rural | 1,879（37.08%） |
| **Region** |  |
| Northern China | 1,672（33.00%） |
| Southern China | 3,395（67.00%） |
| **Income** |  |
| Low | 2,527（49.87%） |
| High | 2,540（50.13%） |
| **Mother’s and Own Education** |  |
| stably high | 252（4.97%） |
| stably low | 1,814（35.80%） |
| generational change | 3,001（59.23%） |
| **Father’s and Own Education,** n(%) |  |
| stably high | 801（15.81%） |
| stably low | 1,729（34.12%） |
| generational change | 2,537（50.07%） |

Table S6. Environmental characteristics during the follow-up period

| **Variables** | **Total（N=5,067）** |
| --- | --- |
| Temperature,℃,mean±SD | **14**·**72 ± 4**·**10** |
| Humidity,%,mean±SD | **72**·**52 ± 4**·**81** |
| PM2.5,μg/m³,mean±SD | 48·49 ± 12·30 |
| Note:Temperature,Humidity,PM2.5: **mean value during the follow-up period.** | |

Table S7. Comparison of Fit Statistics for (LCGA) in Intrinsic Capacity Trajectories (1–5 Classes)

|  | **loglik** | **npm** | **BIC** | **Entropy** | **X.class1** | **X.class2** | **X.class3** | **X.class4** | **X.class5** |
| --- | --- | --- | --- | --- | --- | --- | --- | --- | --- |
| **Latent class growth analysis** | | | | | | | | | |
| 1-class | -35090·27 | 4 | 70214·66 | - | 100·00 |  |  |  |  |
| 2-classes | -34029·42 | 8 | 68127·08 | 0·83 | 86·09 | 13·91 |  |  |  |
| 3-classes | -33664·15 | 12 | 67430·67 | 0·86 | 11·13 | 81·71 | 7·16 |  |  |
| 4-classes | -33296·79 | 16 | 66730·06 | 0·89 | 6·69 | 3·28 | 78·25 | 11·78 |  |
| 5-classes | -33009·68 | 20 | 66189·97 | 0·87 | 71·82 | 7·76 | 2·84 | 13·64 | 3·95 |
| **Growth Mixture Models, random intercept** | | | | | | | | | |
| 1-class | -34558·93 | 5 | 69160·51 | - | 100·00 |  |  |  |  |
| 2-classes | -33889·40 | 10 | 67864·10 | 0·79 | 12·31 | 87·69 |  |  |  |
| 3-classes | -33396·05 | 15 | 66920·06 | 0·83 | 12·73 | 81·09 | 6·18 |  |  |
| 4-classes | -32879·39 | 20 | 65929·39 | 0·83 | 5·01 | 71·38 | 15·08 | 8·53 |  |
| 5-classes | -32733·01 | 25 | 65679·28 | 0·78 | 61·81 | 16·72 | 5·82 | 6·02 | 9·63 |
| **Growth Mixture Models, random intercept and slope** | | | | | | | | | |
| 1-class | -34334·32 | 10 | 68753·95 | - | 100·00 |  |  |  |  |
| 2-classes | -32620·17 | 15 | 65368·29 | 0·65 | 45·53 | 54·47 |  |  |  |
| 3-classes | -32048·97 | 20 | 64268·54 | 0·70 | 21·04 | 24·10 | 54·86 |  |  |
| 4-classes | -31801·45 | 25 | 63816·15 | 0·72 | 37·38 | 10·07 | 12·45 | 40·10 |  |
| 5-classes | -31700·99 | 30 | 63757·89 | 0·73 | 5·96 | 14·23 | 29·01 | 8·23 | 42·57 |

Table S8. Distribution of Intrinsic Capacity Trajectories and Baseline Characteristics among middle aged and old people

| **Variables** | **Total**  **(n = 5,067)** | **High and Stable IC**  **(n = 631)** | **High and slowly decline IC(n = 1,894)** | **Moderate and rapidly decline IC (n = 2,032)** | **Low and rapidly decline IC (n = 510)** | **P** |
| --- | --- | --- | --- | --- | --- | --- |
| **Socio-demographic** |  |  |  |  |  |  |
| **Sex**, n(%) |  |  |  |  |  | <.001 |
| Female | 2,548 (50%) | 256 (41%) | 880 (46%) | 1,089 (54%) | 323 (63%) |  |
| Male | 2,519 (50%) | 375 (59%) | 1,014 (54%) | 943 (46%) | 187 (37%) |  |
| **Age,** n(%) |  |  |  |  |  | <.001 |
| ≥70(years) | 985 (19%) | 27 (4%) | 230 (12%) | 509 (25%) | 219 (43%) |  |
| 50-59(years) | 2,420 (48%) | 452 (72%) | 1,061 (56%) | 808 (40%) | 99 (19%) |  |
| 60-69(years) | 1,662 (33%) | 152 (24%) | 603 (32%) | 715 (35%) | 192 (38%) |  |
| **Marital status**, n(%) |  |  |  |  |  | <.001 |
| married | 4,428 (87%) | 593 (94%) | 1,733 (92%) | 1,705 (84%) | 397 (78%) |  |
| unmarried | 639 (13%) | 38 (6%) | 161 (8%) | 327 (16%) | 113 (22%) |  |
| **Mother’s and Own Education,** n(%) |  |  |  |  |  | <.001 |
| generational change | 3,001 (59%) | 427 (68%) | 1,195 (63%) | 1,131 (56%) | 248 (49%) |  |
| stably high | 252 (5%) | 83 (13%) | 115 (6%) | 50 (2%) | 4 (1%) |  |
| stably low | 1,814 (36%) | 121 (19%) | 584 (31%) | 851 (42%) | 258 (50%) |  |
| **Father’s and Own Education,** n(%) |  |  |  |  |  | <.001 |
| generational change | 2,537 (50%) | 307 (49%) | 991 (52%) | 1,007 (50%) | 232 (45%) |  |
| stably high | 801 (16%) | 207 (33%) | 354 (19%) | 208 (10%) | 32 (6%) |  |
| stably low | 1,729 (34%) | 117 (18%) | 549 (29%) | 817 (40%) | 246 (48%) |  |
| **Urbanization**, n(%) |  |  |  |  |  | <.001 |
| rural | 1,879 (37%) | 354 (56%) | 754 (40%) | 628 (31%) | 143 (28%) |  |
| urban | 3,188 (63%) | 277 (44%) | 1,140 (60%) | 1,404 (69%) | 367 (72%) |  |
| **Income,** n(%) |  |  |  |  |  | <.001 |
| high | 2,540 (50%) | 481 (76%) | 1,110 (59%) | 818 (40%) | 131 (26%) |  |
| low | 2,527 (50%) | 150 (24%) | 784 (41%) | 1,214 (60%) | 379 (74%) |  |
| **Region,** n(%) |  |  |  |  |  | <.001 |
| Northern China | 1,672 (33%) | 166 (26%) | 562 (30%) | 738 (36%) | 206 (40%) |  |
| Southern China | 3,395 (67%) | 465 (74%) | 1,332 (70%) | 1,294 (64%) | 304 (60%) |  |
| **GDP per capita,** n(%) |  |  |  |  |  | <.001 |
| high | 2,682 (53%) | 411 (65%) | 1,039 (55%) | 963 (47%) | 269 (53%) |  |
| low | 2,385 (47%) | 220 (35%) | 855 (45%) | 1,069 (53%) | 241 (47%) |  |
| **Individual health-related factors** |  |  |  |  |  |  |
| **Comorbidity,** n(%) |  |  |  |  |  | <.001 |
| No | 4,533 (89%) | 602 (95%) | 1,748 (92%) | 1,790 (88%) | 393 (77%) |  |
| Yes | 534 (11%) | 29 (5%) | 146 (8%) | 242 (12%) | 117 (23%) |  |
| **Fall,** n(%) |  |  |  |  |  | <.001 |
| No | 4,920 (97%) | 617 (98%) | 1,860 (98%) | 1,976 (97%) | 467 (92%) |  |
| Yes | 147 (3%) | 14 (2%) | 34 (2%) | 56 (3%) | 43 (8%) |  |
| **Physical risk score,** Mean±SD | 1.43 ± 0.93 | 1·32 ± 0·86 | 1·35 ± 0·91 | 1·49 ± 0·96 | 1·61 ± 0·95 | <.001 |
| **Environmental Factor** |  |  |  |  |  |  |
| **Cooking Fuel,** n(%) |  |  |  |  |  | <.001 |
| clean | 2,541 (50%) | 472 (75%) | 1,061 (56%) | 845 (42%) | 163 (32%) |  |
| Non-clean | 2,526 (50%) | 159 (25%) | 833 (44%) | 1,187 (58%) | 347 (68%) |  |
| **Temperature,℃,mean±SD** | 14.72 ± 4.10 | 15.18 ± 4.17 | 15.02 ± 3.98 | 14.42 ± 4.12 | 14.23 ± 4.21 | <.001 |
| **Humidity,℃,mean±SD** | 72.52 ± 4.81 | 73.03 ± 5.16 | 72.83 ± 4.85 | 72.22 ± 4.72 | 71.95 ± 4.41 | <.001 |
| **PM2.5,μg/m³,mean±SD** | 48.49 ± 12.30 | 49.93 ± 12.90 | 49.03 ± 12.34 | 48.16 ± 12.02 | 45.99 ± 12.15 | <.001 |
| P values represent overall comparisons among the four trajectory groups based on ANOVA or chi-square tests. Pairwise comparisons were not performed to avoid excessive multiple testing. | | | | | | |
| F: ANOVA, χ²: Chi-square test, SD: standard deviation | | | | | | |
| **Physical Factor:**BMI, grip strength, pulmonary function, pulse, pre-hypertension  **Comorbidity:**hypertension,diabetes,depression,asthma,stroke,angina,chronic lung diseases  **Temperature, Humidity, PM2.5: Mean value during the follow-up period**  **for father’s (mother’s) and own education:**  **generational change represents** parents had less than primary education, while the participant had at least completed primary school;  **stably high means both parents and the participant had at least completed primary school;**  **stably low** means both parents and the participant had less than primary education. | | | | | | |

Table S9. Preliminary multicollinearity diagnostics of all candidate variables before model selection

| **Term** | **VIF** | **VIF_CI_low** | **VIF_CI_high** | **SE_factor** | **Tolerance** | **Tolerance_CI_low** | **Tolerance_CI_high** |
| --- | --- | --- | --- | --- | --- | --- | --- |
| Fall | 2.667 | 2.553 | 2.789 | 1.633 | 0.375 | 0.358 | 0.392 |
| Comorbidity | 4.491 | 4.280 | 4.715 | 2.119 | 0.223 | 0.212 | 0.234 |
| PM_2.5_ | 4.538 | 4.325 | 4.765 | 2.130 | 0.220 | 0.210 | 0.231 |
| Sex | 5.176 | 4.929 | 5.439 | 2.275 | 0.193 | 0.184 | 0.203 |
| GDP per capita | 7.154 | 6.801 | 7.527 | 2.675 | 0.140 | 0.133 | 0.147 |
| Urbanizaiton | 8.849 | 8.407 | 9.317 | 2.975 | 0.113 | 0.107 | 0.119 |
| Income | 9.687 | 9.201 | 10.203 | 3.112 | 0.103 | 0.098 | 0.109 |
| Cooking Fuel | 15.140 | 14.364 | 15.960 | 3.891 | 0.066 | 0.063 | 0.070 |
| Age | 23.581 | 22.358 | 24.874 | 4.856 | 0.042 | 0.040 | 0.045 |
| Region | 26.429 | 25.055 | 27.882 | 5.141 | 0.038 | 0.036 | 0.040 |
| Matital status | 39.217 | 37.165 | 41.386 | 6.262 | 0.025 | 0.024 | 0.027 |
| Father's Education & Own Education | 63.891 | 60.530 | 67.441 | 7.993 | 0.016 | 0.015 | 0.017 |
| Physical risk score | 9.316 | 8.850 | 9.811 | 3.052 | 0.107 | 0.102 | 0.113 |
| Temperature | 93.390 | 88.465 | 98.593 | 9.664 | 0.011 | 0.010 | 0.011 |
| Mother's Education & Own Education | 101.996 | 96.614 | 107.680 | 10.099 | 0.010 | 0.009 | 0.010 |
| Humidity | 757.741 | 717.586 | 800.146 | 27.527 | 0.001 | 0.001 | 0.001 |

Table S10. Multicollinearity diagnostics of variables included in the multinomial logistic regression model

| **Term** | **VIF** | **VIF_CI_low** | **VIF_CI_high** | **SE_factor** | **Tolerance** | **Tolerance_CI_low** | **Tolerance_CI_high** |
| --- | --- | --- | --- | --- | --- | --- | --- |
| Fall | 2.619 | 2.508 | 2.739 | 1.618 | 0.382 | 0.365 | 0.399 |
| Comorbidity | 4.060 | 3.872 | 4.260 | 2.015 | 0.246 | 0.235 | 0.258 |
| PM_2.5_ | 3.008 | 2.876 | 3.149 | 1.734 | 0.332 | 0.318 | 0.348 |
| Sex | 2.676 | 2.561 | 2.799 | 1.636 | 0.374 | 0.357 | 0.390 |
| GDP per capita | 3.543 | 3.383 | 3.715 | 1.882 | 0.282 | 0.269 | 0.296 |
| Urbanizaiton | 5.320 | 5.065 | 5.591 | 2.306 | 0.188 | 0.179 | 0.197 |
| Income | 5.226 | 4.977 | 5.492 | 2.286 | 0.191 | 0.182 | 0.201 |
| Cooking Fuel | 6.604 | 6.281 | 6.947 | 2.570 | 0.151 | 0.144 | 0.159 |
| Age | 3.975 | 3.791 | 4.170 | 1.994 | 0.252 | 0.240 | 0.264 |
| Matital status | 3.843 | 3.666 | 4.031 | 1.960 | 0.260 | 0.248 | 0.273 |
| Father's Education & Own Education | 5.880 | 5.596 | 6.183 | 2.425 | 0.170 | 0.162 | 0.179 |
| Physical risk score | 2.873 | 2.748 | 3.007 | 1.695 | 0.348 | 0.333 | 0.364 |
| Temperature | 3.619 | 3.454 | 3.794 | 1.902 | 0.276 | 0.264 | 0.290 |
| Mother's Education & Own Education | 5.723 | 5.447 | 6.016 | 2.392 | 0.175 | 0.166 | 0.184 |
| Humidity | 3.697 | 3.528 | 3.877 | 1.923 | 0.270 | 0.258 | 0.283 |

Table S11. Results of sensitivity analyses

| **Group** | **Variables** | **Model A** | | | **Model B** | | | **Model C** | | |
| --- | --- | --- | --- | --- | --- | --- | --- | --- | --- | --- |
|  |  | **OR_HSDI** | **OR_MRDI** | **OR_LRDI** | **OR_HSDI** | **OR_MRDI** | **OR_LRDI** | **OR_HSDI** | **OR_MRDI** | **OR_LRDI** |
| Socio-demographic factors | Age | 1·95(1·66, 2·28) | 3·37(2·86, 3·96) | 6·44(5·27, 7·87) | - | - | - | 1.89 (1.61, 2.22) | 3.15 (2.68, 3.71) | 5.79 (4.73, 7.10) |
|  | Sex | 1·44(1·19, 1·74) | 1·95(1·60, 2·39) | 2·93(2·24, 3·83) | - | - | - | 1.44 (1.19, 1.74) | 1.94 (1.58, 2.37) | 2.86 (2.18, 3.75) |
|  | Marital status | 0·90(0·61, 1·32) | 0·59(0·40, 0·86) | 0·58(0·38, 0·90) | - | - | - | 0.88 (0.60, 1.29) | 0.56 (0.38, 0.82) | 0.53 (0.34, 0.82) |
|  | Mother's and Own Education | 1·20(0·93, 1·55) | 1·26(0·96, 1·66) | 1·44(0·94, 2·20) | - | - | - | 1.19 (0.92, 1.54) | 1.24 (0.94, 1.64) | 1.37 (0.89, 2.12) |
|  | Father's and Own Education | 1·26(1·02, 1·55) | 1·70(1·35, 2·14) | 1·87(1·31, 2·67) | - | - | - | 1.28 (1.04, 1.57) | 1.75 (1.39, 2.21) | 2.04 (1.41, 2.94) |
|  | Urbanizaiton | 0·85(0·65, 1·13) | 0·95(0·70, 1·28) | 1·75(1·12, 2·74) | - | - | - | 0.84 (0.64, 1.11) | 0.92 (0.68, 1.25) | 1.60 (1.01, 2.53) |
|  | Income | 1·40(1·07, 1·83) | 2·21(1·68, 2·91) | 4·02(2·78, 5·82) | - | - | - | 1.41 (1.08, 1.84) | 2.21 (1.68, 2.92) | 4.11 (2.82, 5.99) |
|  | GDP per capita | 1·19(0·95, 1·48) | 1·32(1·05, 1·67) | 0·99(0·73, 1·34) | - | - | - | 1.20 (0.96, 1.50) | 1.39 (1.10, 1.76) | 1.11 (0.81, 1.52) |
| Individual health-related factors | Comorbidity | - | - | - | 2.13 (1.41, 3.21) | 3.90 (2.61, 5.85) | 9.61 (6.16, 14.98) | 2.02 (1.32, 3.08) | 3.56 (2.33, 5.45) | 8.15 (5.07, 13.10) |
|  | Fall | - | - | - | 0.69 (0.36, 1.30) | 0.99 (0.54, 1.83) | 2.78 (1.45, 5.35) | 0.62 (0.32, 1.18) | 0.84 (0.44, 1.61) | 2.25 (1.11, 4.57) |
|  | Physical risk score | - | - | - | 1.05 (0.95, 1.17) | 1.22 (1.10, 1.35) | 1.41 (1.23, 1.61) | 1.01 (0.89, 1.11) | 1.09 (0.98, 1.22) | 1.18 (1.02, 1.37) |
| Environmental Factors | Cooking Fuel | 0·58(0·43, 0·79) | 0·39(0·29, 0·54) | 0·19(0·12, 0·29) | 0.39 (0.31, 0.48) | 0.21 (0.17, 0.25) | 0.12 (0.09, 0.15) | 0.57 (0.42, 0.77) | 0.37 (0.27, 0.51) | 0.17 (0.11, 0.26) |
|  | Temperature | 1·02(0·99, 1·05) | 1·01(0·98, 1·03) | 0·98(0·95, 1·02) | 1.03 (1.01, 1.06) | 1.02 (1.01, 1.05) | 1.02 (0.99, 1.06) | 1.02 (1.01, 1.05) | 1.01 (0.98, 1.04) | 0.99 (0.95, 1.03) |
|  | Humidity | 0·99(0·96, 1·01) | 0·97(0·95, 0·99) | 0·99(0·95, 1·02) | 0.99 (0.97, 1.01) | 0.97 (0.95, 1.01) | 0.97 (0.94, 1.01) | 0.98 (0.96, 1.01) | 0.97 (0.95, 1.01) | 1.01 (0.96, 1.03) |
|  | PM_2.5_ | 1·01(0·73, 1·36) | 0·94(0·68, 1·30) | 1·10(0·72, 1·67) | 1.07 (0.80, 1.43) | 1.11 (0.83, 1.49) | 1.69 (1.17, 2.43) | 1.01 (0.73, 1.37) | 0.99 (0.71, 1.38) | 1.18 (0.77, 1.82) |

Table S12. Multicollinearity diagnostics of predictors used in the machine learning analysis

| **Term** | **VIF** | **VIF_CI_low** | **VIF_CI_high** | **SE_factor** | **Tolerance** | **Tolerance_CI_low** | **Tolerance_CI_high** |
| --- | --- | --- | --- | --- | --- | --- | --- |
| Sex | 2.695 | 2.580 | 2.819 | 1.642 | 0.371 | 0.355 | 0.388 |
| Mother's Education & Own Education | 5.760 | 5.483 | 6.055 | 2.400 | 0.174 | 0.165 | 0.182 |
| Father's Education & Own Education | 5.902 | 5.617 | 6.205 | 2.429 | 0.169 | 0.161 | 0.178 |
| Marital status | 3.859 | 3.682 | 4.048 | 1.965 | 0.259 | 0.247 | 0.272 |
| Urbanizaiton | 5.589 | 5.321 | 5.875 | 2.364 | 0.179 | 0.170 | 0.188 |
| Income | 5.245 | 4.994 | 5.511 | 2.290 | 0.191 | 0.181 | 0.200 |
| GDP per capita | 3.669 | 3.502 | 3.847 | 1.916 | 0.273 | 0.260 | 0.286 |
| Age | 4.047 | 3.860 | 4.246 | 2.012 | 0.247 | 0.236 | 0.259 |
| Comorbidity | 4.080 | 3.892 | 4.281 | 2.020 | 0.245 | 0.234 | 0.257 |
| Fall | 2.622 | 2.511 | 2.742 | 1.619 | 0.381 | 0.365 | 0.398 |
| Cooking Fuel | 6.616 | 6.293 | 6.959 | 2.572 | 0.151 | 0.144 | 0.159 |
| BMI | 2.650 | 2.537 | 2.771 | 1.628 | 0.377 | 0.361 | 0.394 |
| Pulmonary Function | 2.731 | 2.614 | 2.857 | 1.653 | 0.366 | 0.350 | 0.383 |
| Pulse | 2.931 | 2.803 | 3.068 | 1.712 | 0.341 | 0.326 | 0.357 |
| Pre-hypertension | 2.575 | 2.466 | 2.692 | 1.605 | 0.388 | 0.371 | 0.406 |
| Grip strength | 3.748 | 3.577 | 3.930 | 1.936 | 0.267 | 0.254 | 0.280 |
| Humidity | 3.967 | 3.785 | 4.162 | 1.992 | 0.252 | 0.240 | 0.264 |
| Temperature | 3.704 | 3.535 | 3.884 | 1.925 | 0.270 | 0.257 | 0.283 |
| PM_2.5_ | 3.090 | 2.954 | 3.236 | 1.758 | 0.324 | 0.309 | 0.339 |

Note: Predictors include all variables from the multinomial logistic regression, with the behavioral and physical risk scores decomposed into their constituent indicators prior to model fitting.
